# StomaYOLO: A Lightweight Maize Phenotypic Stomatal Cell Detector Based on Multi-Task Training

**DOI:** 10.3390/plants14132070

**Published:** 2025-07-06

**Authors:** Ziqi Yang, Yiran Liao, Ziao Chen, Zhenzhen Lin, Wenyuan Huang, Yanxi Liu, Yuling Liu, Yamin Fan, Jie Xu, Lijia Xu, Jiong Mu

**Affiliations:** 1 College of Information Engineering, Sichuan Agricultural University, Ya’an 625000, China; 202206324@stu.sicau.edu.cn (Z.Y.); 202308391@stu.sicau.edu.cn (Z.L.); 202205661@stu.sicau.edu.cn (W.H.); 2 Maize Research Institute, Sichuan Agricultural University, Chengdu 611130, China; 202207147@stu.sicau.edu.cn (Z.C.); 202406802@stu.sicau.edu.cn (Y.L.); 202406837@stu.sicau.edu.cn (Y.L.); 202205660@stu.sicau.edu.cn (Y.F.); jie_xu@sicau.edu.cn (J.X.); 3 College of Mechanical and Electrical Engineering, Sichuan Agricultural University, Ya’an 625000, China; 18908161812@163.com; 4 College of Law, Sichuan Agricultural University, Ya’an 625000, China

**Keywords:** maize stoma, YOLO, multi-task training, precision agriculture

## Abstract

Maize (*Zea mays* L.), a vital global food crop, relies on its stomatal structure for regulating photosynthesis and responding to drought. Conventional manual stomatal detection methods are inefficient, subjective, and inadequate for high-throughput plant phenotyping research. To address this, we curated a dataset of over 1500 maize leaf epidermal stomata images and developed a novel lightweight detection model, StomaYOLO, tailored for small stomatal targets and subtle features in microscopic images. Leveraging the YOLOv11 framework, StomaYOLO integrates the Small Object Detection layer P2, the dynamic convolution module, and exploits large-scale epidermal cell features to enhance stomatal recognition through auxiliary training. Our model achieved a remarkable 91.8% mean average precision (mAP) and 98.5% precision, surpassing numerous mainstream detection models while maintaining computational efficiency. Ablation and comparative analyses demonstrated that the Small Object Detection layer, dynamic convolutional module, multi-task training, and knowledge distillation strategies substantially enhanced detection performance. Integrating all four strategies yielded a nearly 9% mAP improvement over the baseline model, with computational complexity under 8.4 GFLOPS. Our findings underscore the superior detection capabilities of StomaYOLO compared to existing methods, offering a cost-effective solution that is suitable for practical implementation. This study presents a valuable tool for maize stomatal phenotyping, supporting crop breeding and smart agriculture advancements.

## 1. Introduction

Maize, one of the world’s top three crops, has significantly influenced global food security, environmental sustainability, and smallholder economies over the past two decades [1]. The specialized stomatal complex found in the epidermal tissue of maize leaves, comprising guard cells and accessory cells, serves as a crucial gateway for regulating gas exchange and photosynthesis between the maize plant and its surroundings [2]. Maize can adjust its gas exchange capacity by either reducing stomatal size or increasing stomatal density to enhance its response to drought conditions [3,4]. Therefore, the precise identification of stomata in maize leaves holds substantial theoretical and practical importance for investigating the regulatory mechanisms governing maize stomatal development, physiological reactions to drought stress, and genetic enhancement of crop yield.

Recent years have witnessed a surge in the application of cutting-edge deep learning and computer vision techniques, particularly advancements in algorithms like convolutional neural networks and target detection, to the realm of plant stomatal phenotype detection. Xie et al. [5] successfully employed optical topography in conjunction with Mask Region-based Convolutional Neural Network (Mask R-CNN) to automatically detect epidermal cells and characterize stomata on maize leaves. This approach unveiled the genetic associations between stomatal traits and pinpointed Quantitative Trait Loci (QTLs). Aono et al. [6] utilized a Deep Convolutional Neural Network (DCNN) with a sliding-window mechanism to efficiently classify and identify maize stomata, achieving a classifier accuracy of nearly 97.1% in identifying stomatal regions, offering an automated solution for stomatal research. Jayakody et al. [7] introduced an enhanced Mask R-CNN method incorporating a Feature Pyramid Network (FPN) and statistical filter to achieve the precise instance segmentation of stomata. By mitigating color bias through preprocessing, this method attained an accuracy of 95.10% across 16 datasets, representing a 7% improvement in Intersection over Union (IoU) over conventional techniques. Liang et al. [8] developed the StomataScorer system, integrating deep learning and enhanced computer vision (CV) models to automatically identify and accurately score stomatal features. The system demonstrated a mean absolute percentage error (MAPE) ranging from 0.02% to 6.34%, significantly enhancing detection efficiency and accuracy. Gibbs et al. [9] conducted a comprehensive review of deep learning applications in stomatal analysis, highlighting the training of models such as YOLO and Mask R-CNN on labeled image data using CNN structures for effective stomatal detection, segmentation, and feature extraction. Zhang et al. [10] proposed enhancements to the U-Net architecture by integrating dual symmetric decoding paths, hybrid dilation convolution, and an attention mechanism, substantially boosting multi-scale feature extraction capabilities and enabling the precise segmentation of stomatal regions. The improved U-Net network achieved IoU, PA, and MPA metrics of 83.73%, 98.71%, and 92.16%, respectively, optimizing both accuracy and efficiency.

Despite the progress made by existing methods in stomatal detection, challenges persist in detecting microscopic stomatal datasets due to their small size and the high computational complexity of deploying high-precision models on lightweight devices. The primary difficulty in Small Object Detection in a micro-scale cell dataset lies in the small target size, hindering effective information extraction by the model. To address the bottleneck of Small Object Detection, researchers have proposed innovative solutions in recent years. Wang et al. [11] achieved accurate segmentation by introducing a deep learning process based on UNet++ and entropy-based-masking indicator kriging (IK-EBM), utilizing the UNet++ model for boundary and small target segmentation through multi-scale feature integration and optimized jump connection. Li et al. [12] introduced the YOLOSR-IST method, which incorporates super-resolution preprocessing into the enhanced YOLOv5 architecture, enhancing performance in infrared remote sensing Small Object Detection by reducing leakage rate through the coordinate attention mechanism and high-resolution feature map optimization. P. Liu et al. [13] proposed the YoLoWaternet (YWnet) method, integrating a convolutional block attention module (CBAM), skip residual C3 (SRC3), and Channel Relation Feature Pyramid Network (CR-FPN) modules within the YOLOv5 framework. By combining decoupling head and EIoU loss, this method significantly enhances underwater image detection accuracy and generalization performance. To improve Small Object Feature Extraction, the auxiliary training method in Multi-Task Learning can be utilized to support Small Object Detection training by associating it with larger objectives, which has proven effective. Recent research has shown significant progress in Multi-Task Learning. Wang et al. [14] introduced the cross-task feature enhancement (CTFE) strategy, which reconstructs detection features, aligning them with segmentation features through the feature harmonization module (FHM), and optimizes boundary segmentation by combining it with the asymmetrical feature-sharing and fusing module (AFSFM). This strategy enhances multi-task robust sensing for pepper detection, segmentation, and stem segmentation, notably improving model performance in unstructured environments. Li et al. [15] proposed a multi-tasking weed detection framework based on YOLOv5, comparing the performance of YOLO-High-Resolution Net (HRNet) and YOLO-Pose in weed and jointed stem detection and achieving efficient identification in grassy environments through parameter-sharing. Zhang et al. [16] introduced the YOLO multi-scale (YOLOMS) model, optimizing the YOLOv5s backbone network with the re-parameterized Visual Geometry Group (RepVGG), utilizing the Focal-EIoU loss function, integrating mango detection and fruit stalk segmentation multitasking, and enhancing detection efficiency through feature-sharing. B. Liu et al. [17] proposed a Multi-Task Learning Framework for the Multi-Task Co-Optimization of Tomato Disease Classification and Severity Prediction to enhance model performance through knowledge deconstruction, mutual learning, and knowledge integration strategies.

High-precision models excel in various tasks but are limited in terms of their deployment on mobile devices due to their large parameter scales. Knowledge distillation, an effective method for compressing models, has emerged as a crucial research direction for lightweighting models. This approach involves training lightweight student models to learn soft labels or intermediate features from a teacher model, thereby reducing model complexity while preserving performance. Liu et al. [18] highlighted that knowledge distillation facilitates model compression by transferring knowledge between teacher–student networks, enabling lightweight networks to maintain their detection performance on UAV edge devices for precision agriculture applications. Kang et al. [19] introduced a lightweight single-stage detection model based on the attention mechanism, which maintains accuracy through multi-scale feature fusion and knowledge distillation techniques to enhance the efficiency of rice pest detection. Hu et al. [20] enhanced YOLOv5s to develop a lightweight disease detection model, leveraging Faster-C3 and CoordConv for optimized feature extraction and incorporating channel knowledge distillation to enhance maize leaf disease detection accuracy while reducing parameter count. Gong et al. [21] enhanced YOLOv5s with a MobileNetV3 backbone network, integrated CBAM and Distance–IoU (DloU) for non-maximum suppression (NMS) optimization, and applied knowledge distillation for efficient and accurate maize seedling navigation line detection. Liang et al. [22] proposed the citrus picking-point localization (CPPL) algorithm based on an improved YOLOv8 for efficient and accurate citrus picking-point localization, utilizing knowledge distillation and model pruning techniques with YOLOv8l guiding the training of YOLOv8n.

Building upon the aforementioned analysis, we advocate for an optimized model that leverages the YOLOv11 object detection model, the most recent iteration in the YOLO series, as its primary framework [23]. This model incorporates techniques such as Small Object Detection, Multi-Task Learning, and knowledge distillation to enhance the accuracy of maize stomata detection. The primary contributions of this study are as follows:

1. In this study, a dataset specifically designed for detecting epidermal stomata in maize was developed.
2. This study introduces a target detection model that integrates Small Object Detection, Multi-Task Learning, and knowledge distillation techniques, offering both high accuracy and a lightweight design that is suitable for implementation on a microscope to observe maize epidermal stomata.
3. In this study, we introduced a novel assisted training detection approach utilizing Multi-Task Learning to facilitate the localization and identification of small stomata objects. This method involves establishing a structural hierarchy of maize epidermal tissues and leverages prominent features of large-scale epidermal cells.

## 2. Materials and Methods

### 2.1. Datasets

#### 2.1.1. Dataset Collection

The maize stomatal microscopy data utilized in this research were sourced from the Maize Research Institute at Sichuan Agricultural University. Researchers selected mature leaves from healthy maize plants and prepared samples using the epidermal peeling technique. Intact stomatal structures were obtained by gently pressing the lower leaf epidermis with transparent adhesive tape. Subsequently, the samples underwent glutaraldehyde fixation, ethanol gradient dehydration, and critical point drying, followed by spray-coating with a gold film to enhance imaging quality. Imaging was conducted using a field emission scanning electron microscope (FE-SEM) at 5 kV acceleration voltage and a working distance of 8–10 mm to achieve nanoscale resolution, enabling the clear visualization of stomatal guard cell surface ultrastructure, including cuticle ornamentation and microfibril arrangement. All images were captured in greyscale mode at 6.3× magnification, 10 μm scale, and a pixel resolution of 1360 × 1024. They were saved in 16-bit TIFF format to preserve the full dynamic range. A total of 491 electron microscopy images of maize stomatal cells were obtained for analysis.

#### 2.1.2. Data Processing

**Figure 1:**
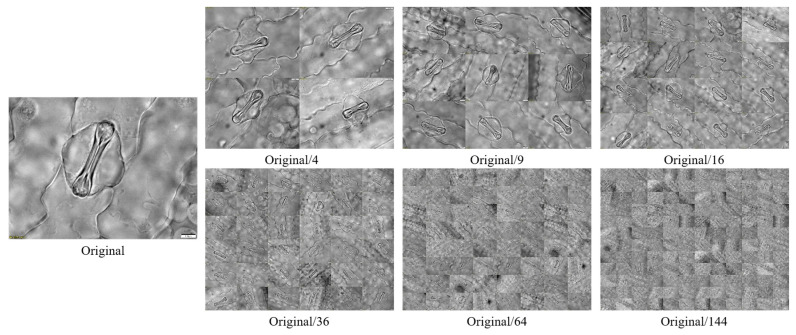
Simulation of stomatal arrangement at different magnifications using the splicing technique.

The maize stomatal dataset analyzed in this study predominantly comprises single-cell samples with a homogeneous background, potentially constraining the applicability of neural network models trained on such data. To improve the models’ generalization capacity and practical utility, we employed image stitching to amalgamate samples. We generated and annotated maize stomatal cell images at various microscope magnifications (depicted in Figure 1; “Original/4” denotes a simulated scenario with a 4:1 scale reduction from the original configuration) to more accurately replicate the diverse conditions encountered in real-world detection settings.

**Figure 2:**
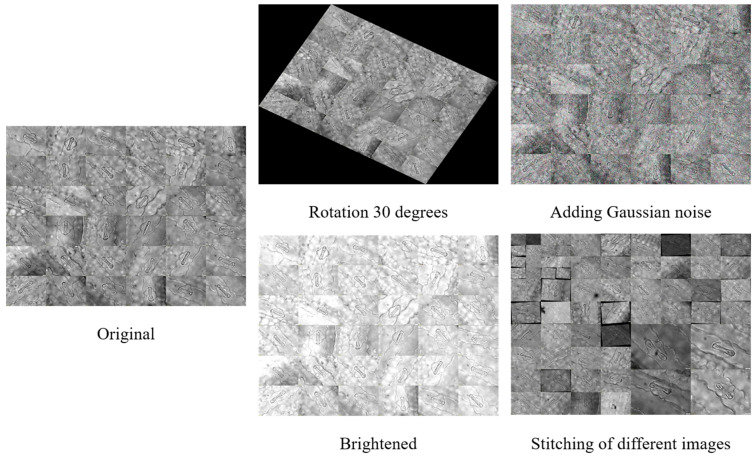
Presentation of some images in the dataset after data enhancement.

To improve dataset diversity and model generalization, we implemented data augmentation techniques including random rotation, brightness adjustments, image combination, splicing, and noise addition. This process yielded over 1500 images for the experimental dataset, with a subset exemplified in Figure 2. The dataset was partitioned using a 7:2:1 ratio for model training and performance evaluation.

### 2.2. Methodology

#### 2.2.1. The Network Architecture of StomaYOLO

**Figure 3:**
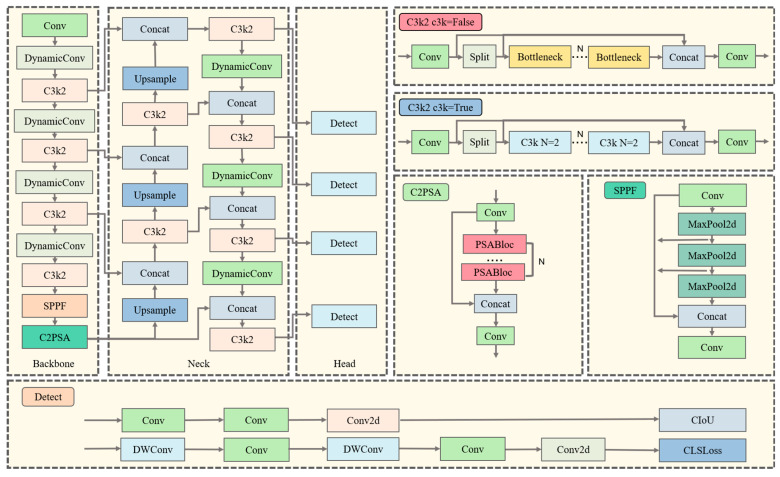
StomaYOLO network structure diagram.

The StomaYOLO model closely follows the algorithmic framework of YOLOv11 from the YOLO algorithm series. Notably, we integrated the dynamic convolution module in place of the standard convolution in YOLOv11, incorporated the Small Object Detection layer P2 in the network architecture, and leveraged auxiliary task training in Multi-Task Learning, along with knowledge distillation during training. These modifications significantly enhanced the network’s feature extraction capabilities and improved the alignment of detection boxes with the maize stomatal aperture edge. The specific network structure of StomaYOLO is illustrated in Figure 3 [24,25].

#### 2.2.2. Multi-Task Learning

**Figure 4:**
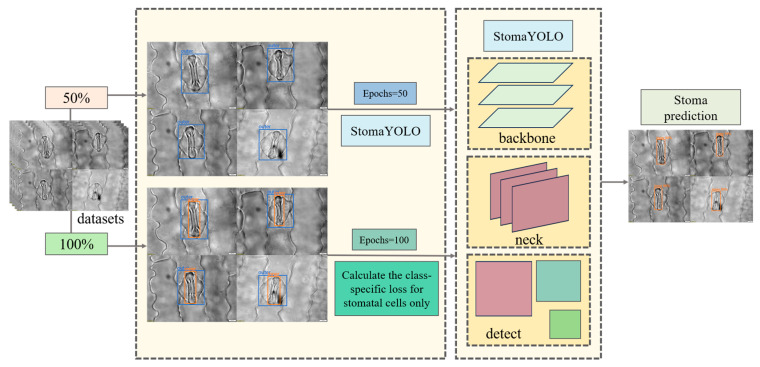
Auxiliary task training steps in Multi-Task Learning.

When detecting stomatal cells, the target cell’s outer layer is surrounded by a larger layer of cells, posing a challenge due to the small size of stomatal cells, which hinders effective feature extraction by the model. To address this issue, we employed auxiliary task training within a Multi-Task Learning framework to enhance model performance. Initially, we conducted 50 rounds of pre-training by randomly selecting 50% of samples from the training and validation sets to create a pre-training dataset comprising solely the labeled information of the outer cells. This pre-training phase aims to enable the model to accurately localize the stomatal cell region during forward propagation, thereby facilitating subsequent feature extraction from stomatal cells. Subsequently, formal training was carried out using the complete training and validation sets, where input images are annotated for both outer-layer cells and stomatal cells. This approach allows the model to acquire a more comprehensive understanding of the field of view. However, during backpropagation, only the stomatal cell loss is computed to ensure that model optimization focuses solely on precise stomatal cell detection. The detailed training process is illustrated in Figure 4.

The initial loss function optimizes the prediction errors for both epidermal and stomatal cells without discrimination, leading to partial gradient updates being allocated to enhance epidermal cell detection accuracy during backpropagation. To prioritize the recognition performance of stomatal cells, we suggest a mask-driven optimization approach. This method involves creating a binary mask based on the original hard labels, represented mathematically as Equation (1):

(1) $$M_{i,j}=\left\{\begin{array}{c} 1\;,\;if\;y_{i,j}^{label}=inner\\ 0\;,\;Otherwise \end{array}\right.$$

Let *M* denote the mask matrix, $y_{i,j}^{label}$ represent the class, and (*i*, *j*) indicate the horizontal and vertical coordinates within the matrix.

Next, we compute the initial binary cross-entropy (BCE) loss for all positions using Equation (2):

(2) $${BCE}_{total}=-\frac{1}{N}\sum_{i,j}\left[y_{i,j}\cdot log(p_{i,j})+(1-y_{i,j})\cdot log(1-p_{i,j})\right]$$

where $p_{i,j}$ represents the model’s predicted probability at position (*i*, *j*), $y_{i,j}$ represents the ground truth label at position (*i*, *j*), and N denotes the total number of pixels.

We train the model by utilizing the specific loss function tailored for stomatal cells, denoted as Equation (3).

(3) $$L_{stima}=\frac{\sum_{i,j}\left[BCE(p_{i,j},y_{i,j})\cdot M_{i,j}\right]}{max(\sum_{i,j}M_{i,j},1)}$$

In our ablation analysis, we extensively examined the impact of the Multi-Task Learning (MTL) framework on model performance. Our experimental findings indicate that the MTL-based model outperforms the traditional single-task baseline model (YOLOv11) significantly. This improvement is mainly attributed to its enhanced feature extraction capabilities, with our suggested large-target-guided feature learning mechanism notably boosting the quality of feature representation for small targets. Quantitative assessments demonstrate a 2.2% increase in mean average precision (mAP) on the test dataset, providing definitive evidence of the effectiveness of the Multi-Task Learning approach.

#### 2.2.3. Small Object Detection Layer

In high-magnification microscopy images, stomatal cells often present as small objects with limited feature information, posing challenges for detection. To address this issue, we propose the integration of a specialized Small Object Detection layer P2, within the network architecture. Positioned at a shallower depth in the network, the Small Object Detection layer P2 operates at a higher feature map resolution, enabling the preservation of intricate spatial details. Leveraging the refined features extracted from the Small Object Detection layer P2 enhances the model’s capacity to discern the morphological characteristics of small-scale targets, thereby facilitating the more precise capture of details pertaining to small objects. This enhancement equips the network with an improved ability to delineate features relevant to stomatal cells in microscopic imagery. The architecture of the enhanced network is delineated in Figure 3.

In the subsequent ablation analysis, we thoroughly examined the impact of integrating a Small Object Detection layer on model performance. Our experimental findings indicate that the model augmented with this supplementary layer exhibits a notable performance enhancement compared to the baseline YOLOv11. This improvement primarily arises from the reinforced multi-scale feature-fusion capability, where our proposed Small Object Detection layer P2 adeptly captures intricate features from shallower feature maps, thereby significantly enhancing the model’s capacity to detect small-scale objects. Quantitative assessments demonstrate a 2.9% increase in mean average precision (mAP) on the test dataset, decisively affirming the effectiveness of this architectural refinement.

#### 2.2.4. DynamicConv

The primary application scenario for this model involves detecting stomatal cells in maize leaf microscopic images within a laboratory setting, necessitating high inference efficiency. To address this, we incorporate the DynamicConv mechanism. Dynamic convolution operates by dynamically selecting or combining various convolution kernels for each input sample, enabling the network to adjust its parameters based on input features.

In traditional neural networks, a typical convolutional layer operates on input features, denoted as *X*, by applying a weight tensor W, as shown in Equation (4):

(4) Y=X∗W

In this context, *Y* signifies the output, * denotes the convolution operation, and for simplicity, we exclude the bias term. It is important to recognize that a fully connected layer can be conceptualized as a specific instance of a convolutional operation using 1 × 1 kernels.

The design philosophy of DynamicConv aims to enable the implementation of intricate network structures without incurring significant computational costs. This objective is realized by employing a parameter augmentation mechanism that increases the model’s capability (see Equation (5)):

(5) W′=f(W)

The function f must meet two essential criteria: (1) computational efficiency and (2) a substantial improvement in model capacity through the incorporation of more trainable parameters. DynamicConv operationalizes Equation (6) as follows:

(6) $$W\prime =\sum_{i=1}^{M}\alpha_{i}W_{i}$$

where $W_{i}$ denotes the i-th convolutional weight tensor and $\alpha_{i}$ represents its corresponding dynamic coefficient. These coefficients are sample-specific and generated through an input-dependent multilayer perceptron (MLP). The generation process first applies global average pooling to input *X*, producing a compact representation vector, which is then processed by a two-layer MLP with softmax activation Equation (7):

(7) α=softmax(MLP(Pool(X)))

**Figure 5:**
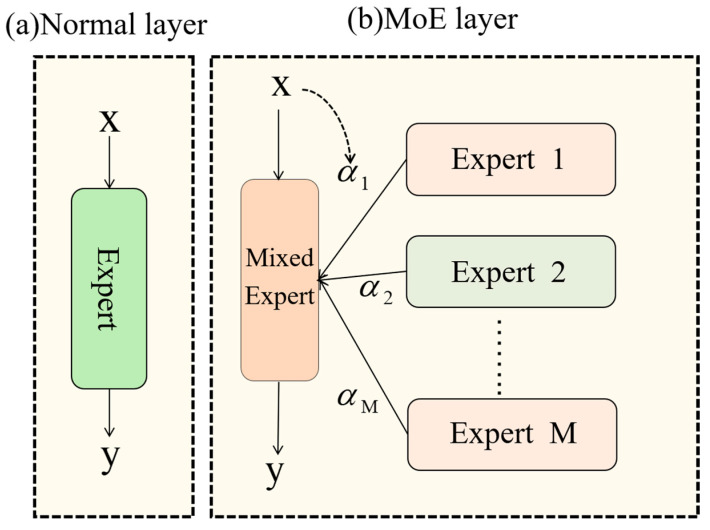
Working principle of DynamicConv.

DynamicConv, functioning as a proficient dynamic convolution operator (shown as the MoE layer in Figure 5), enables a substantial parameter capacity increase while minimizing computational expenses via kernel-sharing and dynamic combination. This feature renders it well-suited for implementing intricate network structures with limited computational resources. Thus, DynamicConv exhibits robust adaptability to the practical application scenarios examined in this investigation.

In our ablation analysis, we systematically assess the influence of DynamicConv on model performance. Our experimental findings indicate that integrating DynamicConv leads to notable enhancements in both accuracy and computational efficiency compared to the baseline YOLOv11. This improvement is attributed to DynamicConv’s adaptive kernel mechanism, which dynamically adjusts convolutional weights to optimize feature extraction by selectively enhancing discriminative features and suppressing redundant ones. As a result, the model achieves higher precision while reducing computational costs. Quantitative evaluation reveals that this approach enhances the model’s mean average precision (mAP) by 0.2% on the test dataset while decreasing Giga Floating-point Operations Per Second (GFLOPS) by 1.6. Comparative assessments with other lightweight models further demonstrate that the finalized model incorporating DynamicConv outperforms these models in terms of computational efficiency, thereby affirming the efficacy of integrating DynamicConv.

#### 2.2.5. Knowledge Distillation

**Figure 6:**
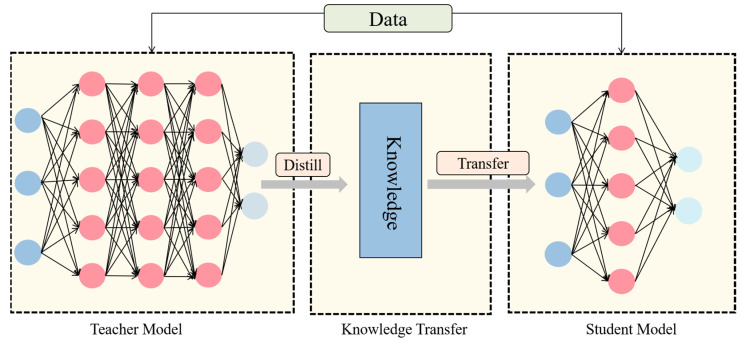
Core steps of knowledge distillation.

This model is primarily utilized for identifying stomatal cells in maize leaves through electron microscopy in a laboratory setting, with a focus on enhancing detection accuracy and deployment efficiency. Traditional approaches often enhance detection performance by escalating model intricacy, which in turn escalates computational demands and poses challenges regarding efficient deployment on constrained devices like electron microscopes. To address this issue, we propose integrating a knowledge distillation technique during model training to enhance detection accuracy without augmenting model complexity. Knowledge distillation facilitates the student model in refining decision boundaries by transferring the insights from the high-capacity teacher model to the lightweight student model through soft labels generated by the teacher model [26]. Figure 6 delineates the fundamental stages of knowledge distillation.

During the training of our model, we utilize Masked Generative Distillation Loss (MGDLoss) for knowledge distillation. In contrast to traditional methods that focus on the direct replication of the teacher model’s feature responses by the student model, our approach based on MGDLoss reframes feature imitation as a feature generation challenge. This novel strategy mandates that the student model recreates the teacher model’s higher-order features within its constrained representation capability, which proves to be highly advantageous in situations characterized by restricted sample diversity [27].

Given that images exhibit local similarity and neighboring pixels share information, the MGDLoss leverages pixels to reconstruct the entire feature map. Denoting the l-th feature map of the teacher and student as T and S, respectively, a random mask is initially applied to cover the student’s l-th feature, as shown in Equation (8):

(8) $$M_{i,j}^{l}=\left\{\begin{array}{c} 0,\;if\;R_{i,j}^{l}<\lambda \\ 1,\;Otherwise \end{array}\right.$$

where $R_{i,j}^{l}$ is a random number in (0,1), *i* and *j* are the horizontal coordinates of the feature maps, respectively, λ is a hyperparameter that represents the ratio of the masks, and the *l*-th feature map is covered by the *l*-th random mask. The MGDLoss method utilizes this mask to conceal the student’s feature map. The masked student features are then fed into the generative network to produce reconstructed teacher features. Subsequently, the disparity between the generated features and the actual teacher features is assessed to derive the loss value for backpropagation. This process can be mathematically represented by Equations (9) and (10):

(9) $$G(f_{align}(S_{k,i,j}^{l})\cdot M_{i,j}^{l})\to T^{l}$$

(10) $$G(F)=W_{l2}(ReLU(W_{l1}(F)))$$

where *G* denotes the projection layer, including two convolutional layers, $W_{l1}$ and $W_{l2}$, and one activation layer, ReLU. Based on the above method, the MGDLoss demonstrated in Equation (11) is obtained:

(11) $$L_{dis}\left(S,T\right)=\sum_{l=1}^{L}\sum_{k=1}^{C}\sum_{i=1}^{H}\sum_{j=1}^{W}{\left(T_{k,i,j}^{l}-G(f_{align}(S_{k,i,j}^{l})\cdot M_{i,j}^{l})\right)}^{2}$$

Here, *L* represents the total number of distillation layers, while *C*, *H*, and *W* refer to the dimensions of the feature maps. *S* and *T* represent the features of the students and teachers, respectively.

For the final loss, MGDLoss trains all models using the following total loss (Equation (12)):

(12) $$L_{all}=L_{original}+\alpha \cdot L_{diss}$$

where $L_{original}$ is the original loss of the model in all tasks and α is a hyperparameter to balance the loss.

In essence, the precise protocol for employing MGDLoss in knowledge distillation entails the following steps: initially, the student model processes inputs to derive its feature representation and predicted outputs, while the teacher model processes the same inputs to obtain their feature representations. Subsequently, the loss of the student model for the original task and the distillation loss are computed, and a weighted combination of these components yields the total loss. This total loss is then utilized for back-propagation to update the parameters of the student model, resulting in the optimized student model.

In our comparative experiments assessing knowledge distillation, we thoroughly examined the impact of the MGDLoss-based distillation technique on model performance. Our results indicate a significant enhancement in performance when employing the MGDLoss method compared to both the baseline model and models trained using alternative distillation loss functions. This improvement is primarily attributed to MGDLoss’s capacity to augment the model’s learning capabilities through label-softening. Specifically, the teacher model refines supervision signals by softening the probability distribution, enabling the student model to capture inter-class correlation features and thereby enhancing its representation-learning proficiency. Quantitative analysis validates the efficacy of the MGDLoss-based distillation framework, demonstrating a 1.1% increase in test-set mAP without escalating model complexity.

### 2.3. Experimental Environment

The experimental setup utilizes an Ubuntu (64-bit) operating system(developed by Canonical Ltd., London, United Kingdom) running on a 12-core Intel(R) Xeon(R) Platinum 8255C CPU @ 2.50 GHz processor (Intel Corporation, Santa Clara, CA, USA), with an RTX 3080 GPU. The PyTorch(version 1.8.1) open-source deep learning framework serves as the development environment, with Python 3.8, a CUDA version of 11.1, and 43 GB of computer memory(Corsair, Fremont CA, USA).

### 2.4. Evaluation Indicators

This study assessed the maize stomatal detection model’s performance using seven metrics: precision, mAP, recall, FPS, parameters, GFLOPS, and weight. The definitions of these parameters used in the formulas for calculating the evaluation indicators are presented in Table 1.

TP represents the number of samples correctly predicted as positive by the model, FP refers to the number of samples incorrectly predicted as positive by the model, FN is the number of samples incorrectly predicted as negative by the model, and TN indicates the number of samples correctly predicted as negative by the model.

Precision indicates the probability that a sample predicted to be positive is actually positive. The specific calculation formula is shown in Equation (13).

(13) $$\;Precision=\frac{TP}{TP+FP}$$

AP denotes the integral from P-index to R-index. mAP refers to the average of AP values for all categories. The specific calculation formula is shown in Equations (14) and (15).

(14) $$AP=\int_{0}^{1}P(R)dR$$

(15) $$mAP=\frac{1}{K}\sum_{i=1}^{K}AP_{i}$$

Recall denotes the proportion of individuals correctly judged by the model to be in the positive category out of all individuals that are actually in the positive category. The specific calculation formula is shown in Equation (16).

(16) $$Recall=\frac{TP}{TP+FN}$$

FPS (Frames Per Second) is a key performance metric used to quantify the number of image frames processed per second by a model or system, which directly reflects the inference efficiency of the model. A higher FPS value indicates that the model can process more image frames per unit time and that its computational performance is stronger. The specific formula for this metric is shown in Equation (17), where Inference_Time_per_Frame represents the time (in seconds) required by the model to complete inference on a single image frame.

(17) $$FPS=\frac{1}{Inference\_Time\_per\_Frame}$$

GFLOPS, short for Giga Floating-point Operations Per Second, serves as a fundamental metric for assessing a model’s computational efficiency by quantifying the billions of floating-point operations executed per second during its reasoning process, directly mirroring the model’s computational complexity. Conversely, model weights denote the storage capacity of its parameters, typically quantified in MB or GB. Reduced GFLOPS and weights values signify lower computational and storage demands, yielding a more streamlined model structure. In contrast, higher GFLOPS and weights values indicate increased computational and storage overhead, resulting in a more intricate model architecture.

Parameters are crucial indicators of model complexity, reflecting the aggregate count of all trainable parameters within the model. While a higher number of parameters can enhance the theoretical representation of the model, it can also escalate computational expenses and the likelihood of overfitting. Assessing the parameter count is a common method for gauging model magnitude, and by fine-tuning this count, a harmonious equilibrium between model efficacy and computational effectiveness can be attained.

## 3. Results

### 3.1. Ablation Experiments

To thoroughly assess the efficacy of the optimization algorithms utilized in this study and to comprehensively evaluate the performance enhancement of the integrated algorithm for maize stomatal detection, a series of ablation experiments were conducted. These experiments were designed to investigate the individual contributions of each component to the overall performance by systematically removing or substituting key elements of the algorithm. Table 2 presents the metric values obtained from the ablation experiments, where √ denotes the inclusion of a method in the network and × denotes its exclusion. The incorporation of auxiliary task training, in conjunction with the Small Object Detection head, led to improvements in mAP, precision (P), and recall (R); however, it significantly impacted computational speed. Subsequently, the integration of novel convolutional techniques aimed to strike a balance between enhancing detection accuracy and performance while maintaining computational efficiency. The results depicted in the table highlight the optimal performance of the model through the combined application of the three methods. Specifically, Model 8 achieved an mAP of 90.7%, with a reduction in GFLOPS of 1.8 compared to Model 4, which yielded a similar mAP. This suggests that the computational efficiency of the model is notably enhanced through the amalgamation of the three methods.

### 3.2. Comparative Experiments

To establish the superior efficiency and performance of the algorithmic framework proposed in this study, a series of comparative experiments was conducted. Various representative algorithmic frameworks were selected to ensure a comprehensive and unbiased comparison.

#### 3.2.1. Comparative Experiments on Knowledge Distillation

**Figure 7:**
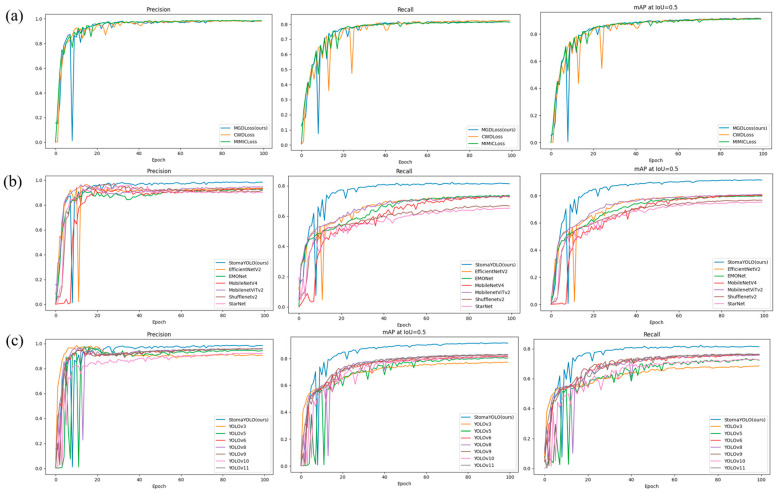
Visualization of the results of the different comparison experiments. (**a**) Comparison results of different knowledge distillation loss functions, (**b**) comparison of lightweight backbone networks, and (**c**) performance evaluation across object detection models.

To evaluate the impact of various knowledge distillation loss functions on model performance and determine the optimal detection model, we assessed three common distillation loss functions: Channel-Wise Distillation Loss (CWDLoss), MGDLoss, and Multi-Instance Mutual Information Loss (MIMILoss). The comparative experiments were carried out using a consistent teacher–student model framework and training configuration to ensure a fair evaluation of each loss function’s knowledge transfer efficacy. The results presented in Table 3 and Figure 7a illustrate the metrics obtained from the knowledge distillation experiments. Our findings indicate that employing MGDLoss as the distillation loss function yielded the most effective detection performance, achieving a mean average precision (mAP) value of 91.8%.

#### 3.2.2. Lightweight Backbone Comparison Experiments

To assess the overall performance of the proposed model in terms of accuracy and efficiency, this study conducted comparative experiments with several popular lightweight backbone networks, such as MobileNetV4, ShuffleNetv2, and EfficientNetv2. The experiments utilized a standardized dataset and training setup to ensure impartial evaluation, focusing on computational complexity (quantified in GFLOPS and parameter count) and detection accuracy (quantified in mAP). Detailed results of the lightweight backbone comparison experiments are presented in Table 4 and Figure 7b. The findings indicate that, compared to MobileNetViTv2, the proposed model enhances mAP by 11.8% and FPS by 23.5 with similar computational requirements, while reducing GFLOPS by 1.4. Furthermore, in comparison to other lightweight models, the proposed model achieves an average mAP improvement of approximately 10%, with comparable FPS and GFLOPS. These outcomes underscore the notable benefits of the proposed model in terms of accuracy and efficiency, effectively striking a balance between detection performance and computational resource utilization.

#### 3.2.3. Model Comparison Experiments

**Figure 8:**
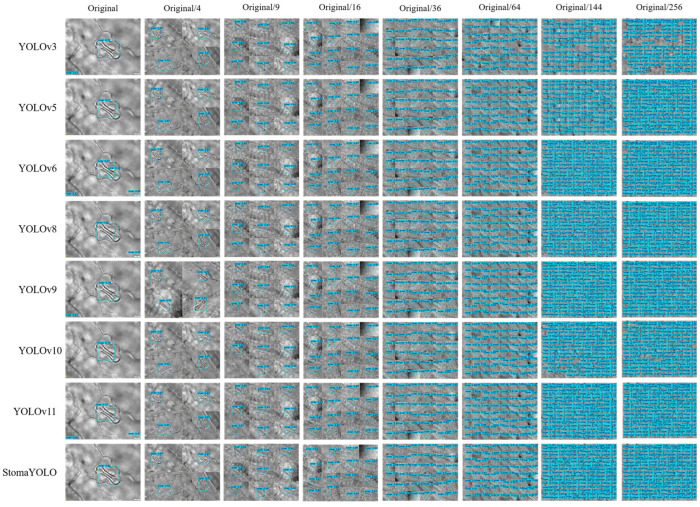
Display of some results of the test sets.

To assess the efficacy and performance superiority of the algorithmic framework proposed in this study, a series of comparative experiments were conducted against several established algorithmic frameworks. The selection of these frameworks aimed to ensure a comprehensive and unbiased evaluation. The outcomes of these experiments, presented in Table 5 and Figure 7c, depict the results and provide a visual representation of the comparative analysis of various target detection methods. The results indicate that the proposed model exhibits an average improvement of approximately 9% in detection accuracy (mAP) compared to the alternative models, while maintaining a moderate to high level of model complexity in terms of parameter count and computational complexity (GFLOPS). Furthermore, as illustrated in Figure 8, the StomaYOLO model demonstrates reduced instances of both detection and missed detection in comparison to other target detection models. These findings underscore the superior equilibrium between efficiency and performance of the proposed model, thereby conferring a substantial overall performance advantage over existing models.

## 4. Discussion

In the context of advancing precision agriculture and plant phenomics, the forefront of agricultural information technology has shifted towards plant microstructure detection technology. Among the various target detection algorithms, the YOLO series models have emerged as prominent due to their superior real-time performance, optimal accuracy–speed balance, and robust scene adaptability. Nonetheless, a critical examination of prior studies reveals potential enhancements are warranted in terms of their accuracy, lightweight design, and detection speed.

This paper introduces StomaYOLO, a novel target detection network designed for identifying epidermal stomatal cells in maize leaves. StomaYOLO surpasses conventional YOLO models by integrating a Small Object Detection Layer (P2) and DynamicConv mechanisms to enhance the fusion of deep and shallow features, thereby improving computational efficiency for real-time, high-precision detection. The P2 layer enhances small object detection by extracting detailed features from shallow maps, while DynamicConv optimizes feature extraction efficiency through adaptive convolutional kernel-sharing and dynamic combination. This approach prioritizes relevant features, leading to a notable 3.4% increase in mean average precision (mAP) compared to the YOLOv11 baseline model, with minimal additional computational cost. During training, we employed auxiliary task training in Multi-Task Learning and knowledge distillation, utilizing a cascade detection method based on multi-scale correlated features to establish the structural hierarchy of maize epidermal tissues. By leveraging the salient features of large-scale epidermal cells, the model accurately localizes and identifies stomata in Small Object Detection. Through knowledge distillation, we enhanced model accuracy without increasing parameters or computations. The experimental results on a self-generated maize epidermal stomatal cell dataset demonstrate that StomaYOLO outperforms other models by 9–10% in mAP with only 8.4 GFLOPS of computation. Unlike traditional machine learning methods requiring manual feature extraction design, this model automates feature extraction through deep learning algorithms, enhancing generalization capabilities without the constraints of manual engineering. With ample training data, this model can be effectively fine-tuned for various plant cell types and applications.

Throughout our investigation, we faced notable challenges and identified key limitations in our study. First, our dataset lacks diversity, particularly in simulating maize epidermal stomatal cells across different magnification levels using image-stitching techniques. While effective to a certain extent, this method still presents noticeable disparities compared to the intricate structures of actual stomatal cells. Second, our dataset predominantly comprises images of healthy cells in the closed state, with few samples depicting alternative stomatal morphologies. This limitation inherently hinders the model’s ability to generalize comprehensively.

## 5. Conclusions

The StomaYOLO model proposed in this study efficiently and accurately detects maize epidermal stomatal cells by creating a specialized dataset and integrating Small Object Detection, Multi-Task Learning, and knowledge distillation techniques. This model excels in detection accuracy, computational efficiency, and ease of deployment. It achieves impressive metrics—91.8% mAP, 98.5% precision, 81.6% recall, and only 8.4 GFLOPS—surpassing many lightweight backbone models in overall performance. The StomaYOLO model represents a valuable tool for investigating maize stomatal development, responses to drought, genetic enhancement, and advancements in precision agriculture and plant microphenotyping. Future research will focus on expanding the dataset with diverse images of maize epidermal cells at varying magnifications and morphologies. Additionally, we plan to explore generative network techniques like the Generative Adversarial Network (GAN) and diffusion family to enhance model generalization.

## Figures and Tables

**Table 1:** Parameters of evaluation indicators.

Confusion Matrix		Predicted Results	
		Positive	Negative
Expected Results	Positive	TP	FN
	Negative	FP	TN

**Table 2:** Results of ablation experiment indicators.

Model	T	D	C	P	R	mAP	FPS	Parameters	GFLOPS	Weight/MB
1	×	×	×	0.958	0.766	0.833	114.8	2,582,542	6.3	5.2
2	√	×	×	0.965	0.77	0.855	112.1	2,582,542	6.3	5.2
3	×	√	×	0.968	0.786	0.862	86.4	2,659,552	10.2	5.6
4	√	√	×	0.977	0.798	0.902	89.8	2,659,552	10.2	5.6
5	×	×	√	0.966	0.771	0.835	83.9	4,588,486	4.7	9.1
6	×	√	√	0.972	0.781	0.867	80.9	4,692,692	8.4	9.4
7	√	×	√	0.969	0.781	0.869	118.1	4,588,486	4.7	9.1
8	√	√	√	0.979	0.807	0.907	82.8	4,692,692	8.4	9.4

T denotes auxiliary task training; D denotes Small Object Detection head; C denotes DynamicConv.

**Table 3:** Knowledge distillation comparison experiment results of indicators.

Model Core	P	R	mAP	FPS	Parameters	GFLOPS	Weight/MB
None (YOLOv11)	0.958	0.766	0.833	114.8	2,582,542	6.3	5.2
CWDLoss	0.981	0.822	0.913	83.1	4,692,692	8.4	9.5
MIMICLoss	0.983	0.813	0.91	78.7	4,692,692	8.4	9.5
**MGDLoss (Ours)**	**0.985**	**0.816**	**0.918**	**81.8**	**4,692,692**	**8.4**	**9.5**

**Table 4:** Results of the indicators of the lightweight backbone comparison experiment.

Model	P	R	mAP	FPS	Parameters	GFLOPS	Weight/MB
MobilenetViTv2	0.945	0.731	0.808	58.3	3,012,583	9.8	6.1
ShuffleNetv2	0.913	0.668	0.768	90.8	1,705,318	4.1	3.6
EMONet	0.926	0.738	0.799	52.0	2,417,793	6.5	5.0
StarNet	0.92	0.728	0.818	75.8	1,895,296	8.1	4.2
EfficientNetv2	0.933	0.728	0.806	88.3	2,086,978	5.3	4.4
MobileNetV4	0.928	0.734	0.807	108.9	2,167,582	5.2	4.5
**Ours**	**0.985**	**0.816**	**0.918**	**81.8**	**4,692,692**	**8.4**	**9.5**

**Table 5:** Results of the metrics from comparative experiments with different target detection models.

Model	P	R	mAP	FPS	Parameters	GFLOPS	Weight/MB
YOLOv3-tiny	0.909	0.683	0.774	411.8	12,128,692	18.9	23.2
YOLOv5	0.945	0.735	0.809	142.9	2,503,334	7.1	5.0
YOLOv6	0.957	0.759	0.818	181.5	4,233,942	11.8	8.7
YOLOv8	0.958	0.757	0.827	155.5	3,006,038	8.1	6.0
YOLOv9	0.958	0.763	0.829	66.5	1,971,174	7.6	4.4
YOLOv10	0.922	0.732	0.814	101.3	2,695,196	8.2	5.5
YOLOv11	0.958	0.766	0.833	114.8	2,582,542	6.3	5.2
**Ours**	**0.985**	**0.816**	**0.918**	**81.8**	**4,692,692**	**8.4**	**9.5**

## Data Availability

The data presented in this study are available on request from the corresponding author. The code that support the findings of this study are openly available in GitHub at https://github.com/yangziqi2003/StomaYOLO (accessed on 5 May 2025).
